# Aesthetic principles access thyroidectomy produces the best cosmetic outcomes as assessed using the patient and observer scar assessment scale

**DOI:** 10.1186/s12885-017-3645-2

**Published:** 2017-09-18

**Authors:** Xiao Ma, Qi-jun Xia, Guojun Li, Tian-xiao Wang, Qin Li

**Affiliations:** 1 Department of Head and Neck, Perking University Cancer Hospital and Institute, 52 Fucheng Road, Haidian District, Beijing, China; 2 Department of General Surgery, PLA Rocket General Hospital, 16 Xinjiekouwai Street, Xicheng District, Beijing, China; 3 Department of Head and Neck Surgery, The University of Texas MD Anderson Cancer Center, 1515 Holcombe Blvd, Houston, TX USA; 4 Department of Epidemiology, The University of Texas MD Anderson Cancer Center, 1515 Holcombe Blvd, Houston, TX USA; 5 Department of Oncology, Beijing Friendship Hospital, Capital Medical University, 95 Yongan Raod, Xicheng District, Beijing, 100050 China; 6 Key Laboratory of Carcinogenesis and Translational Research, Department of Head and Neck, Perking University Cancer Hospital and Institute, Beijing, 100142 China

**Keywords:** Thyroid surgery, Thyroidectomy, Minimally invasive access, Aesthetic principle, POSAS

## Abstract

**Background:**

Thyroid carcinoma (TC) is more likely to occur in young women. The aim of this study was to compare the aesthetic effect of different thyroidectomies.

**Methods:**

One hundred twenty female patients who underwent thyroidectomy were evenly distributed into three groups: conventional access (CA), aesthetic principles access (APA) and minimally invasive access (MIA). The Patient and Observer Scar Assessment Scale (POSAS) was used as the assessment tool for the linear scar.

**Results:**

The patients in the MIA group showed significantly less intraoperative blood loss, less drainage, a shorter scar length and a shorter duration of drainage than those in the CA group and the APA group. However, the operation time of 129.0 min in the MIA group was significantly longer than the 79.6 min in the CA group and the 77.0 min in the APA group. The best aesthetic score, as assessed by the Observer Scar Assessment Scale (OSAS), was obtained in the APA group. The Patient Scar Assessment Scale (PSAS) scores were significantly lower in the APA group and CA group than in the MIA group. Significantly lower objective scar ratings were found in the APA group than in the other two groups.

**Conclusion:**

These results show that APA produced the best surgical outcomes in TC patients, indicating that conventional thyroidectomy can produce an ideal aesthetic result using the principles of aesthetic surgery. Thyroid surgery need not be performed through excessively short incisions for the sake of patient satisfaction with the scar’s appearance.

**Trial registration:**

This clinical trial was retrospectively registered on ClinicalTrials.gov PRS on August 1st,2017 (NCT03239769).

## Background

Thyroid carcinoma (TC), especially differentiated thyroid carcinoma (DTC), is one of the most common malignancies in the head and neck region [1, 2]. The prognosis of DTC is excellent, with a 10-year survival rate greater than 91% [3]. This disease is more likely to occur in young women, who may be concerned about the aesthetic appearance of the scar resulting from the thyroidectomy. Therefore, the pursuit of more favorable aesthetic effects is a priority for thyroid surgeons.

Since the introduction of endoscopic parathyroidectomy by Gagner in 1996 and endoscopic thyroidectomy by Hüscher CS et al. in 1997, new techniques, such as a robotic-assisted transaxillary approach, a video-assisted anterior chest approach and a transoral endoscopic approach, have been reported to improve the cosmetic results [4–7]. Compared with open procedures, these techniques undoubtedly have some advantages, such as faster recovery and scarless incision. However, these innovative procedures present the disadvantages of increased operative time, additional endoscopic instrumentation, and new complications, including brachial plexus injury and external and internal jugular vein, carotid artery or tracheal lesions. Moreover, these procedures cannot ensure the radical resection of thyroid carcinoma as with open access, which is the standard approach for thyroid carcinoma [8].

Even without the assistance of endoscopic instruments, thyroidectomy with an incision between 3 and 3.5 cm long can be performed by a professional endocrine surgeon. A recent cohort study found that incision length may not be critical in decision making for thyroid cancer surgery [9]. Moreover, other head and neck procedures such as oral cavity surgery have shown no improvement in patient satisfaction with lip-splitting mandibulotomy approach versus trans-oral approach [10]. Therefore, the aim of this study was to evaluate and compare the surgical outcomes, aesthetic effects and incision length of different access procedures in patients with DTC.

## Methods

### Patient characteristics and data collection

We conducted a prospective study in patients with DTC at the Department of Head and Neck Surgery at Perking University Cancer Hospital. A total of 120 female patients who underwent surgical treatment for DTC were enrolled in the study from June 2012 to June 2014. All patients were diagnosed with DTC through preoperative fine needle aspiration biopsy pathology. These patients were individually randomly assigned (1:1:1 ratio) into the conventional access group (CA), the aesthetic principles access group (APA) or the minimally invasive access group (MIA). Lobectomy plus ipsilateral central lymph node dissection (CLND) was adopted in each patient. DTC staging [11] was T1N0M0 or T1N1M0. We retrieved the patients’ information, including age, incision length, incision closure procedure, incidence of complications, and cosmetic assessment from their medical records. Patients with other medical diseases, such as diabetes or obesity, a smoking history, a keloid tendency, a history of radiotherapy to the head and neck, or with incomplete information, were excluded. RLN function was evaluated by electronic fiber laryngoscopy 6 months postoperatively. The follow-up time was 12.3 months. The research was reviewed and approved by the Ethics Committee of Peking University Cancer Hospital, and informed consent was obtained from all patients to publish the information/image(s) in an online open-access publication. The study was open-label with no blinding of patients, clinicians, or research staff.

### Surgical procedure

Lobectomy plus CLND was performed by the same surgical team. The patients were divided into the CA group, the APA group and the MIA group.

#### Conventional access thyroidectomy (CA group)

A 4- to 5-cm incision was created, subplatysmal flaps were raised, and the strap muscles were mobilized. Then, the superior pole of the thyroid gland was exposed. Using blunt dissection, the superior pole vessels were isolated and then ligated using No.4 silk suture. The parathyroid glands were identified and preserved with their vascular pedicles. The gland was retracted medially, and the RLN was identified inferiorly and traced to its entrance into the cricothyroid junction with division of the ligament of Berry. Then, the gland was delivered through the surgical incision, and the thyroid isthmus was divided. Finally, CLND was performed. A careful inspection of the wound was performed to avoid homeostasis. The strap muscles were re-approximated with No.1 silk suture. The full-thickness skin was closed with interrupted monofilament, and then a closed suction drainage system was used.

#### Aesthetic principles access thyroidectomy (APA group)

The entire surgical process was similar to that of CA. The key difference focused on the disposal incision using aesthetic principles, which are depicted below. When performing the APA procedure, the incision was protected by Vaseline ointment. Excessive skin traction was avoided to prevent the injury on the skin edge. Bleeding was stanched with a low-power bipolar coagulation device. The surgical field does not have to be pulled in every direction to show the full operation field. When performing the parathyroid preservation procedure, the skin must be pulled only to show the appropriate field to preserve the parathyroid. When closing the midline, the cervical linea alba was closed by continuous sutures with 3–0 absorbable Vicryl sutures. Interrupted sutures of 4–0 Vicryl were used to re-approximate the subcutaneous tissues. The epidermis was fixed with 3 M steri-strip elastic skin closures rather than skin sutures.

#### Minimally invasive access thyroidectomy (MIA group)

With the MIA approach, a shorter incision of between 3 and 4 cm was created. The procedure used the Harmonic scalpel as an auxiliary device. First, the isthmus was divided. Second, the lower pole of the thyroid was dissected from the adipose tissue, and the inferior thyroid vessels were divided close to the thyroid gland for mobilization. The RLN and parathyroid glands were carefully dissected. Third, the superior pole of the thyroid gland was disconnected. Finally, CLND was performed. The closure procedure for the incision was similar to that for APA.

### Aesthetic evaluation tool

**Fig. 1:**
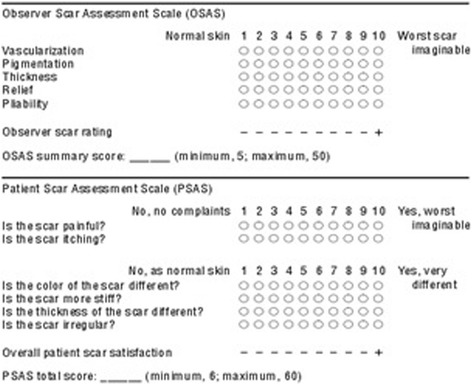
The Patient and Observer Scar Assessment Scale

The Patient and Observer Scar Assessment Scale (POSAS) was used as an assessment tool in our study. The POSAS scale is a reliable and feasible tool for linear scar evaluation [12, 13]. The POSAS included the observer scale and the patient scale. The Observer Scar Assessment Scale (OSAS) score was obtained by the same observer; this scale includes 5 items graded on a 10-point scale with 1 indicating normal skin and 10 indicating the worst scar imaginable. A summary score of 5 indicates normal skin, and a summary score of 50 is the worst possible scar result. The Patient Scar Assessment Scale (PSAS) consists of 6 items. All items are graded by the patient on a 10-point scale; a summary score of 6 to 60 represents the range from normal skin to the worst imaginable scar. After scoring the items, the observer and the patients rated the overall scar appearance on a visual analogue scale corresponding to a 10-point scale (Fig. 1).

### Statistical analysis

The SPSS statistical package (version 19.0; Chicago, IL) was used for all data analysis. For category data, the differences between groups and within groups were analyzed by Chi-square test or the Fisher’s exact test. Continuous values were reported as the mean ± standard deviation (SD). Differences in continuous variables were analyzed by ANOVA or the Student t-test. Additionally, Bonferroni correction was used for multiple comparison. A *P* value of less than 0.05 was considered statistical significant.

## Results

### Patient characteristics

One hundred twenty patients were divided into the conventional access (CA) group, the aesthetic principles access (APA) group and the minimally invasive access (MIA) group, with 40 patients per group. The age distribution of the whole population ranged from 25 to 57 years, and the average age was 37.0 years in the CA group, 35.4 years in the APA group and 37.6 years in the MIA group. There were no significant differences among the three groups. Papillary carcinoma accounted for more than 95% of all cases.

**Fig. 2:**
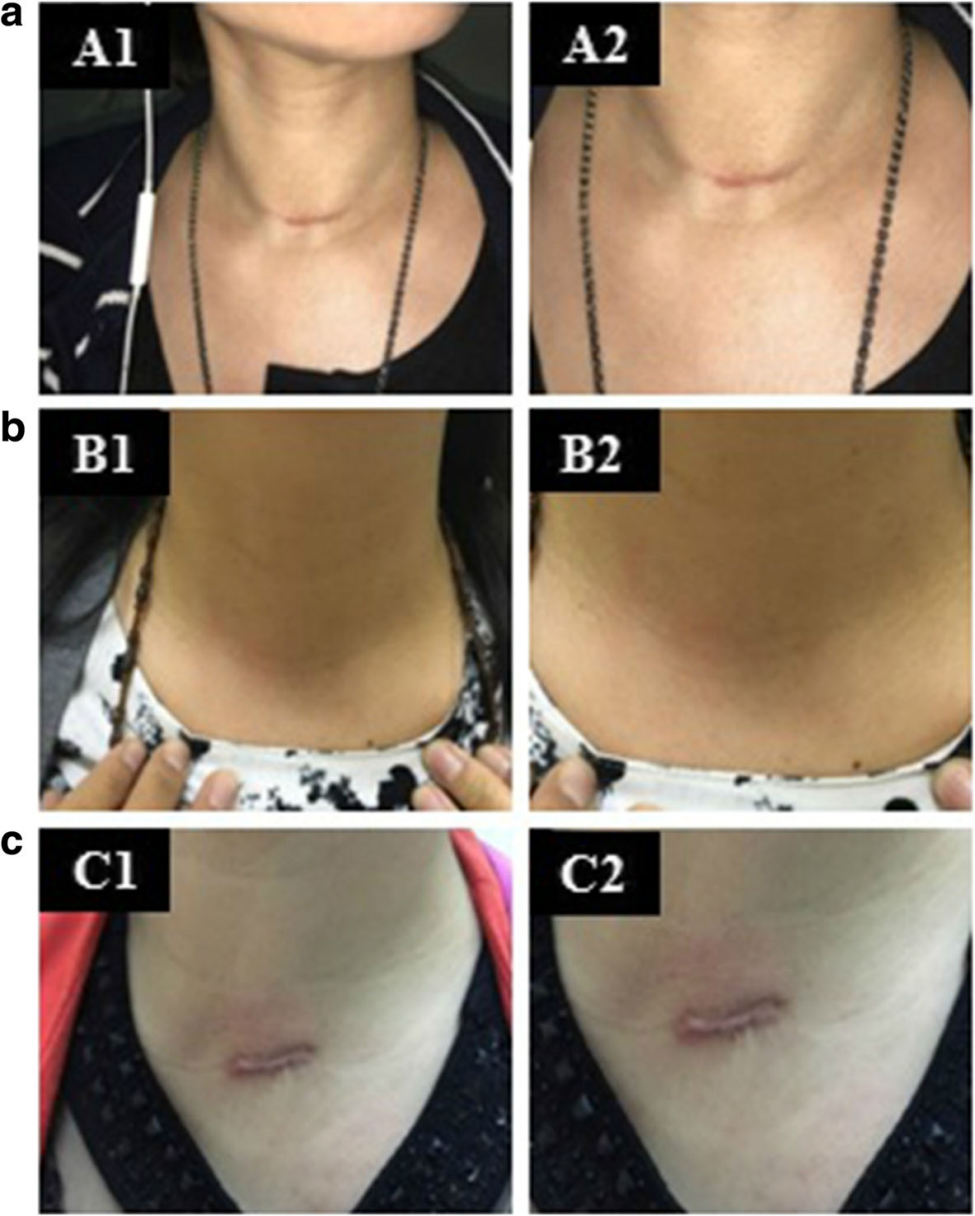
Digital images obtained from the patients after surgery. **a**: Conventional access thyroidectomy (CA); **b**: Aesthetic principles access thyroidectomy (APA); **c**: Minimally invasive access thyroidectomy(MIA)

Digital images obtained from the patients of the three groups are shown in Fig. 2. The best cosmetic effect was seen in patients with the APA approach, and the worst cosmetic effect was seen in patients with the MIA approach. The cosmetic effect of patients receiving the CA approach was between those of the APA approach and MIA approach (Fig. 2).

### Comparison of peri-operative features among the three groups

The operation time of 129.0 min in the MIA group was significantly longer than the 79.6 min in the CA group and the 77.0 min in the APA group (MIA vs. CA, *P* < 0.001; MIA vs. APA, *P* < 0.001; CA vs. APA, *P* = 0.918). The patients in the MIA group showed significantly less intraoperative blood loss (MIA vs. CA, *P* < 0.001; MIA vs. APA, *P* < 0.001; CA vs. APA, *P* = 0.438), significantly less drainage (MIA vs. CA, *P* < 0.001; MIA vs. APA, *P* < 0.001; CA vs. APA, *P* = 0.438), a significantly shorter scar length (MIA vs. CA, *P* < 0.001; MIA vs. APA, *P* < 0.001; CA vs. APA, *P* = 0.999), and a significantly shorter duration of drainage (MIA vs. CA, *P* < 0.001; MIA vs. APA, *P* < 0.001; CA vs. APA, *P* = 0.476) than the CA group and the APA group. However, the latter two groups were not significantly different (Table 1).

**Table 1:** Comparison of peri-operative features among the three groups

Variables	CA	APA	MIA	*P* value
	(*N* = 40)	(*N* = 40)	(*N* = 40)	
Operation time (min)	79.6 ± 15.9	77.0 ± 17.2	129.0 ± 26.3	<0.001
Blood loss (ml)	36.3 ± 15.4	37.2 ± 18.9	29.4 ± 14.7	<0.001
Amount of drainage (ml)	53.7 ± 27.8	55.3 ± 29.8	35.4 ± 16.3	<0.001
Duration of drainage (day)	1.9 ± 0.4	2.1 ± 0.6	1.6 ± 0.5	<0.001
Number of CLND	3.1 ± 0.7	3.2 ± 0.5	3.0 ± 1.2	0.322

*CA* conventional thyroidectomy, *APA* aesthetic principles access thyroidectomy, *MIA* minimally invasive thyroidectomy

### Comparison of the patient and observer assessment scale scores among the three groups

**Fig. 3:**
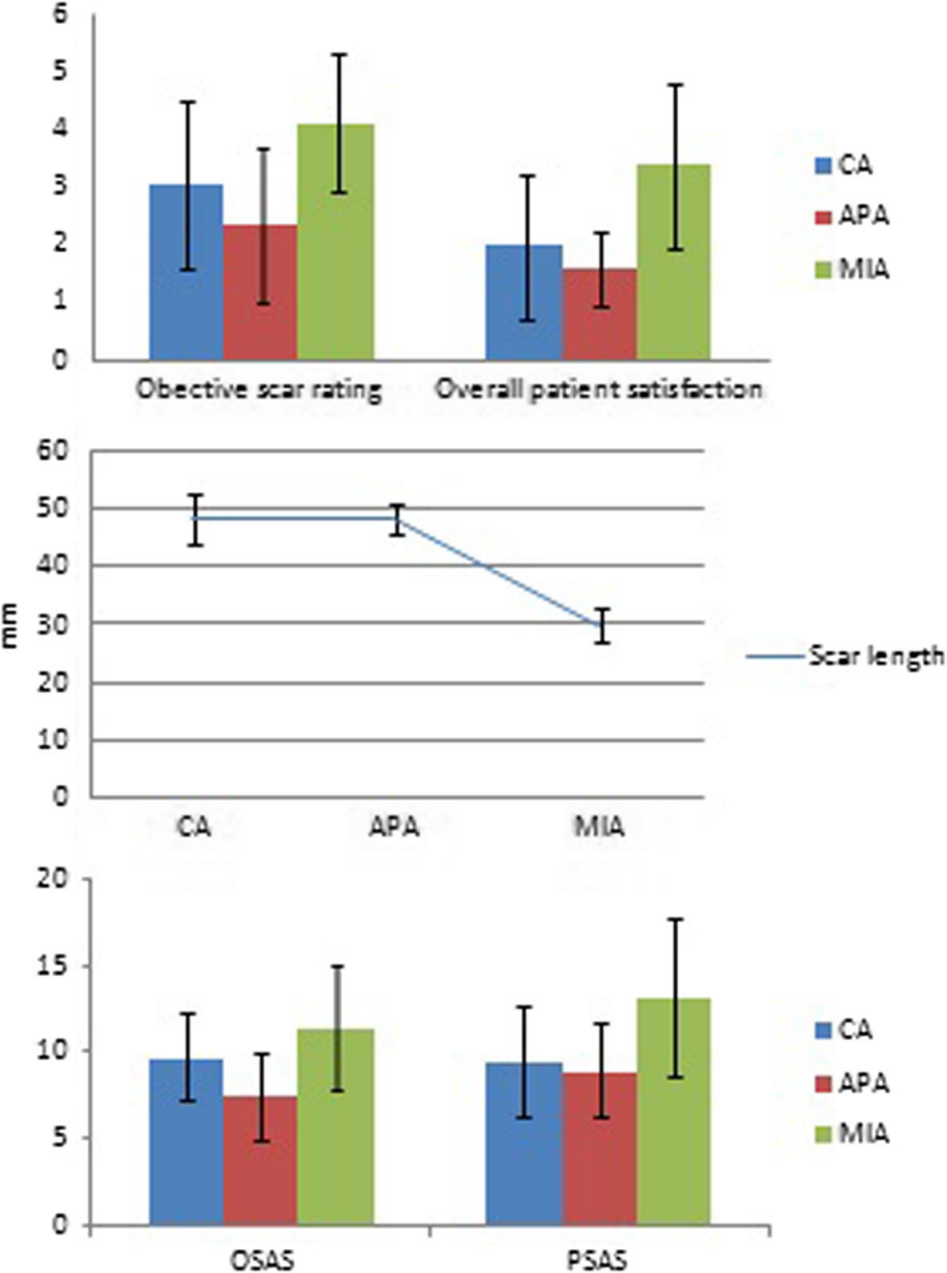
Comparison of the Patient and Observer Assessment Scale scores

Our results showed that cosmetic satisfaction was highest in the APA group, followed by the CA group and then the MIA group. The best aesthetic score was obtained in the APA group using the Observer Scar Assessment Scale (OSAS) (APA vs. CA, *P* < 0.001; APA vs. MIA, *P* < 0.001; CA vs. MIA, *P* = 0.0326). Patient Scar Assessment Scale (PSAS) scores were significantly lower in the APA group and the CA group than that in the MIA group (APA vs. CA, *P* = 0.874; APA vs. MIA, *P* < 0.001; CA vs. MIA, *P* < 0.001). Significantly lower objective scar ratings were found in APA group patients (APA vs. CA, *P* = 0.06; APA vs. MIA *P* < 0.001; CA vs. MIA, *P =* 0.003) than in CA groups. Very small differences were found in overall patient satisfaction and scar length between patients in the APA group and the CA group, and the patients in these two groups showed lower scores than those in the MIA group (satisfaction: APA vs. CA, *P* = 0.323; APA vs. MIA, *P* < 0.001; CA vs. MIA, *P <* 0.001; scar length: APA vs. CA, *P* = 0.999; APA vs. MIA, *P <* 0.001; CA vs. MIA, *P <* 0.001) (Table 2, Fig. 3).

**Table 2:** Comparison of Patient and Observer Assessment Scale scores

Variables	CA (*N* = 40)		APA (*N* = 40)		MIA (*N* = 40)		*P* value
	Mean ± SD	Median (range)	Mean ± SD	Median (range)	Mean ± SD	Median (range)	
Scar length (mm)	48.35 ± 4.29	46(43–56)	48.25 ± 2.65	48(44–55)	30.00 ± 3.00	30(24–35)	<0.001
POSAS score							
OSAS	9.80 ± 2.54	10(6–13)	7.48 ± 2.46	7(5–12)	11.48 ± 3.60	11(6–17)	<0.001
PSAS	9.52 ± 3.18	9(6–16)	8.98 ± 2.75	8(6–14)	13.27 ± 4.56	12(8–21)	<0.001
Objective scar rating	3.05 ± 1.47	3(1–5)	2.35 ± 1.35	2(1–5)	4.10 ± 1.19	4(2–6)	<0.001
Overall patient satisfaction	1.98 ± 1.25	2(1–4)	1.60 ± 0.63	2(1–3)	3.37 ± 1.43	3(1–6)	<0.001

*CA* conventional thyroidectomy, *APA* aesthetic principles access thyroidectomy, *MIA* minimally invasive thyroidectomy, *POSAS* Patient and Observer Scar Assessment Scale, *OSAS* Observer Scar Assessment Scale, *PSAS* Patient Scar Assessment Scale

### Complication assessment

The postoperative complications were observed among the three groups. There was one case of permanent recurrent laryngeal nerve (RLN) palsy in the MIA group, which was confirmed by electronic fiber laryngoscopy examination and manifested as voice hoarseness. No cases were found in the CA group or the APA group. No permanent hypocalcemia was found in any of the patient. One case of bleeding occurred in the CA group, and one case of infection occurred in the MIA group. One case of hematoma occurred in the APA group and one in the MIA group (Table 3).

**Table 3:** Comparison of postoperative complications among the three groups

Variable	CA	APA	MIA
	(*N* = 40)	(*N* = 40)	(*N* = 40)
Permanent RLN palsy	0	0	1
Temporary hypocalcemia	1	0	0
Permanent hypocalcemia	0	0	0
Bleeding	1	0	0
Hematoma	0	1	1
Infection	0	0	1

*CA* Conventional thyroidectomy, *APA* Aesthetic principles disposal of incision, *MIA* Minimally invasive thyroidectomy, *RLN* Recurrent laryngeal nerve

## Discussion

Recent advances in surgery have focused on minimally invasive techniques. The concept of minimally invasive surgery (MIS) was first proposed by Wickham, an English urologist [14]. The goal of MIS is not only to make smaller incisions but also to minimize wound complications, decrease postoperative pain and hospital stays, and attain better aesthetic outcomes. The MIS principle has also been adopted by surgeons focusing on thyroid cancer.

Regardless of which minimally invasive thyroidectomy approach is used, video-assisted techniques and the development of extracervical surgical approaches aim to reduce scarring. Miccoli et al. compared scar satisfaction from video-assisted thyroidectomy, parathyroidectomy and conventional techniques using a non-validated verbal response scale to assess overall patient satisfaction 1 month after surgery. Bellantone also asked patients to rate their overall satisfaction with their scar at 3 and 6 months after surgery and compared the results for video-assisted and conventional thyroidectomies. The results of these two studies showed that smaller neck incisions improved patient satisfaction with scar cosmesis [15, 16]. However, long-term assessment methods were used in other studies, and no significant differences in patient satisfaction were noted between incisions from minimally invasive techniques and those from conventional surgery [17]. The study by Toll EC et al. demonstrated no association between absolute scar length or relative scar length ratio and patient satisfaction at 2–24 months after the conventional approach thyroidectomy. There was also no association found between absolute or relative scar length and satisfaction in female patients [18]. In our study, the follow-up time was more than one year. Although MIA was performed to improve postoperative scars, it led to the worst aesthetic effects as a result. The relationship between scar length and patient satisfaction does not appear to be as certain as previously thought.

Wound healing studies have demonstrated that scars usually develop after 6–8 weeks following re-epithelization, and a period of 6–18 months is required for scar maturation. Healing and remodeling are largely completed by 8–12 months; and scars might be delayed until 1 year for evaluation [19, 20]. Therefore, the observation time is critical to drawing an appropriate conclusion. There are many factors potentially influencing patient satisfaction with scar cosmesis instead of the length of the incision, such as the degree of hypertrophy, keloid formation, pigmentation, and discomfort experienced by patients [18]. Mow et al. showed that the cosmesis of mini-incision total hip replacement scars was inferior to that of standard-incision scars because skin and soft tissue damage were produced by the high retractor pressures, which were needed for exposure using a limited skin incision [21]. When a minimally invasive approach was used, the use of retractors for a longer time to increase exposure was inevitable. Thus, the edges of the wound might be traumatized from the stretching of the surgical wound to remove a gland or perform central lymph node dissection (CLND). These injuries could inevitably affect the aesthetic level of wound healing.

In addition to improvement of incision appearance, decreasing postoperative complications was another principle of the MIS approach. The first credible records of thyroid surgery appeared in the School of Salerno in the thirteenth century, although the techniques consisted simply of the use of cottons and hot irons for hemostasis. The technique of capsular dissection made the conventional access thyroidectomy practical and relatively safe [22–24]. In our study, CA was deemed a reliable method and showed very low postoperative complications, with only one case with bleeding, who required a second hemostasis and one case of temporary asymptomatic hypocalcemia, who was self-healed 5 days after the operation. There was one case of permanent RLN palsy in the MIA group. However, RLN did not occur in the CA group or the APA group. This adverse event might have been caused by the excessively short incision, which led to a poor surgical field and increased risk of damage to important structures, such as the parathyroid glands and RLN, at the cost of a longer operation time. Nevertheless, our current study had some limitations, such as small sample size, all patients from a single-center study. Thus, a large-scale, prospective, multicenter clinical study should be conducted to validate these findings.

## Conclusion

In summary, these results suggest that aesthetic principles access produces the best surgical outcomes in TC patients. Minimally invasive access thyroidectomy demonstrated the highest rate of postoperative complications and the worst aesthetic results, although it has the advantages of less intraoperative blood loss and a reduced scar length. However, conventional thyroidectomy may obtain an ideal aesthetic result using the principles of aesthetic surgery. Head and neck surgeons should pay closer attention to aesthetic principles in thyroidectomy. Indeed, unnecessarily small incisions may cause unsatisfactory results; therefore, thyroid surgery need not be performed through excessively short incisions for the sake of patient satisfaction with the scar’s appearance.

### Clinical practice points

- Thyroid carcinoma (TC), especially differentiated thyroid carcinoma (DTC), is one of the most common malignancies in the head and neck region and this disease is more likely to occur in young women.
- Minimally invasive access thyroidectomy has been applied to solve the cosmetic problems that resulted from conventional thyroidectomy.
- In our study, we found that conventional thyroidectomy may obtain an ideal aesthetic result using the principles of aesthetic surgery.
- The minimally invasive access thyroidectomy demonstrated the highest rate of postoperative complications and the worst aesthetic results, and therefore thyroid surgery need not be performed through excessively short incisions for the sake of patient satisfaction with the scar’s appearance.

## Abbreviations

APA: Aesthetic principles access
CA: Conventional access
DTC: Differentiated thyroid carcinoma
MIA: Minimally invasive access
POSAS: Patient and observer scar assessment scale
TC: Thyroid carcinoma
